# Physiological Responses and the Dust Retention Ability of Different Turfgrass Mixture Ratios Under Continuous Drought

**DOI:** 10.3390/plants14111667

**Published:** 2025-05-30

**Authors:** Junrui Wang, Haimei Li, Dehong Gong, Xiujun Liu, Bingqi Liu, Xiao Guo

**Affiliations:** 1 College of Landscape Architecture and Forestry, Qingdao Agricultural University, Changcheng Road NO. 700, Qingdao 266109, China; 2 College of Environmental and Geographical Sciences, Qingdao University, Qingdao 266071, China

**Keywords:** drought stress, dust stress, mixture ratio, physiological variables

## Abstract

Drought is one of the main environmental disturbances limiting the growth and production of turfgrass in China and around the world. To study the performance under drought conditions of different mixing ratios (*Lolium perenne* L., *Festuca elata* Keng., *Poa pratensis* L.), a water-controlled pot experiment was conducted. The mixing ratios used were 2:3:5, 2:6:2, and 2:2:6 for *Lolium perenne*, *Festuca elata*, and *Poa pratensis*, respectively. The relative water content (RWC), proline (Pro) content, and other physiological and ecological variables of three turfgrass monocultures and their three ratio mixtures (a total of six different treatments) were measured under drought as well as dust stress at various time points. The results revealed that, under drought stress, the dust retention performance of the mixing ratio treatments was better than the monocultures, with the best performance in the 2:6:2 mix and the worst in the *Poa pratensis* monoculture. Additionally, during the 21 days of drought stress, as time increased, the appearance quality (TQ) of the turfgrass gradually declined over time; its RWC gradually decreased; its chlorophyll (Chl) content, peroxidase (POD) activity, and superoxide dismutase (SOD) activity all showed a trend of initially increasing then decreasing; and its soluble sugar (Sol), malondialdehyde (MDA), and Pro content increased continuously. A comprehensive evaluation of physiological and ecological variables, using the membership function method, showed that the six types of turfgrass treatments ranked as follows (from strongest to weakest) in drought resistance: 2:6:2 mix > *Festuca elata* monoculture > 2:3:5 mix > 2:2:6 mix > *Lolium perenne* monoculture > *Poa pratensis* monoculture. The dust retention capability was assessed through quantitative measurements, and the ranking of dust retention amounts in descending order was as follows: *Festuca elata* > 2:6:2 mix > 2:2:6 mix > *Poa pratensis* > *Lolium perenne* > 2:3:5 mix. We conclude that, in practical applications, the degree of drought can be appropriately controlled within a certain range to achieve maximum dust retention benefits from turfgrass.

## 1. Introduction

Due to global warming, arid and semi-arid areas now occupy more than one-third of the world’s land surface, where they account for about 52.5% of China’s land territory [1]. The Qingdao region of China has a warm temperate monsoon climate with an average annual temperature of 12.7 °C, and average annual precipitation of 600–800 mm that is concentrated in summer [2]. Drought, as a complex and gradual natural phenomenon, is one of the most common environmental disasters in the world, characterized by its long duration and extent. As a key biotic component of urban and semi-urban ecosystems, turfgrass has a significant role to play in global carbon cycle research. In the dry season, drought and water scarcity may lead to a decline in turf quality, and some leaves may show wilting, resulting in a reduction in the ecological benefits of turfgrass. Hence, drought has become one of the main environmental factors that limits the normal growth and development of turfgrass and also affects turf quality [3]. According to Chaves et al. [4], when drought stress occurs, the mechanism by which plants resist drought is mainly reflected in their ecological adaptability. Turfgrass was shown to resist drought by enlarging its root system, maintaining a high leaf water-retention capacity, and enhancing its water-use efficiency. Turfgrass was able to respond via changes in biomembrane stability, photosynthesis and chlorophyll regulation, stomatal closure, antioxidant enzyme activity, and osmotic action. Felipe et al. [5] showed that under drought conditions, turfgrass can prevent excessive water loss by regulating its own osmotic regulation, reducing osmotic potential and maintaining its water content to resist the damage caused by this abiotic stress. Toscano et al. [6] imposed drought stress upon two shrubs, finding that plants could resist drought stress in several ways: by reducing photosynthetic activity, enhancing stomatal control, reducing leaf water content, increasing the accumulation of osmotic regulatory substances (such as proline), and activating SOD and catalase (CAT) enzymes. By studying gramineous plants, research by Terashima et al. [7] has shown that the drought resistance of turfgrass is achieved via physiological responses that improve its survival ability under drought stress, that the epidermal characteristics and anatomical structure of turfgrass leaves are a direct reflection of the stage of plant growth and development and level of adaptation to the external environment, and that the drought resistance of turfgrass is closely related to the chlorophyll content and other physiological variables of its leaves.

Research shows that urban plants can effectively capture particulate matter in the atmosphere, serving as an effective measure to mitigate urban particulate pollution [8,9]. Urban plants can reduce emission of surface particulate matter by covering the ground [10]. Meanwhile, the leaves of plants can intercept airborne particulate matter, and enhance the deposition of particulate matter by improving micro-meteorological conditions [11,12], thereby improving the quality of the atmospheric environment. Accordingly, afforestation can effectively capture and fix dust, reducing the amount of dust in the air, which plays a crucial role in improving levels of air quality in urban and semi-urban areas [13], and reduce human exposure to inhalable particulate matter in urban areas. Leaves possess a strong capacity for the adsorption and retention of particulate matter due to their unique surface characteristics [14]; however, the PM retention capacity of plant leaves exhibits interspecies variation [15,16,17,18]. The particulate matter (PM) retention capacity of plant foliage is significantly influenced by the synergistic effects of interspecific leaf morphological characteristics [19,20,21,22], while, under field conditions, the dust deposition on urban vegetation remains dynamically variable due to environmental factors such as drought [23]. For example, Liu et al. [24] showed that drought stress increases the production of cuticular wax, and the thickness of a leaf’s wax layer affects its dust retention ability. Therefore, the dust retention potential of plants is related to the degree of drought they experience. Räsänen et al. [25] found that, under drought, the dust retention ability of spruce (*Picea asperata Mast*) was higher than that under normal water conditions. Similarly, Kong et al. [26] found that drought enhanced the dust retention ability of red osmanthus (*Photinia × fraseri*) leaves under conditions of drought stress.

Garden plants, as one of the main modes of urban and semi-urban area greening, inevitably encounter drought during their cultivation [27,28]. Nyfeler et al. [29] studied mixed communities and found that using different mixing ratios had an important impact on plants’ growth and development as well as their stress resistance. Guo et al. [30] studied vegetation under drought conditions, finding that water is a pivotal factor affecting its growth and development status and various physiological activities, also being a pertinent factor in whether plants could grow normally in a city. The experimental results of perennial herbs by Picotte et al. [31] showed that plant leaf morphology would respond to changes in water availability to adapt to the environment, which mainly involved various characteristics such as growth and development, morphological characteristics, and physiological indexes.

Most existing studies have focused on the response of individual grass species under stress conditions. However, in practical applications, turfgrass is predominantly cultivated through mixed seeding, and its quality directly affects its dust retention capacity [32]. Therefore, this study selects cold-season turfgrass species commonly used in northern regions [33] to investigate their responses to drought conditions through sustained drought stress. The research aims to elucidate their drought resistance mechanisms and, by simulating artificial dust deposition, examine the physiological responses and changes in dust retention capacity of six turfgrass mixtures under varying drought intensities. The study seeks to identify the optimal mixture configuration, selecting combinations with strong drought resistance and high dust retention capacity, thereby providing theoretical and data-driven support for the practical application of mixed-seeding turfgrass in urban greening.

## 2. Materials and Methods

### 2.1. Experimental Materials

Current research mostly focuses on the response of a single grass species to external stress. However, in practical applications, in order to better build lawns, a combination of dominant species, clustered species, and associated species is often adopted. These grass species often need to have the ability to face adverse environments such as soil drought and scarce precipitation, and form communities to jointly resist external stress.

*Lolium perenne*, with its rapid establishment, high yield, and quality, has become an ideal choice for forage production and ecological restoration. Therefore, it is often used in environmental restoration. Moreover, due to its high nutritional content and certain economic value, it is widely used as lawn grass. Its drought resistance is moderate, and it is necessary to ensure sufficient water during the growth period. Ryegrass, due to its fast growth rate and strong tillering ability, performs outstandingly in preventing soil erosion and quickly establishing vegetation coverage. Its resistance to trampling makes it an ideal choice for urban greening and ranch management, especially for degraded land that requires rapid restoration [34]. *Festuca elata* has strong adaptability, outstanding stress resistance, is resistant to trampling and diseases, and does not go into dormancy in summer. It is a grass species suitable for wide promotion and use. It is applicable to use in various lawns such as golf courses, sports fields, landscaping, and soil and water conservation, as well as for slope protection of highways, railways, and river embankments. The stems and leaves of *Poa pratensis* are soft, with good uniformity, density, and smoothness, making it suitable for building various types of lawns. *Poa pratensis* prefers sunlight, is drought-tolerant and cold-resistant, and is not strict about soil requirements. It has strong adaptability and strong covering power, and can be used to create grassland along roadsides or as a plant for road slopes.

In recent years, the expansion of urban landscaping areas has significantly stimulated domestic researchers to continue studying turfgrass species suitable for local climatic conditions. However, the current urban green space coverage remains below 10%, with per capita urban green space even less than 1.6 square meters. The demand for turfgrass is enormous, driven by needs such as landscaping for industrial complexes; sports field construction; soil and water conservation projects along highways, railways, and riverbanks; and desertification control—all requiring large-scale, high-quality turfgrass. These potential economic opportunities have greatly facilitated research related to turfgrass [35].

Therefore, in this study, three common gramineous plants in the northern regions of China were selected as the research objects. They are respectively *Lolium perenne*, *Festuca elata*, and *Poa pratensis* planted by seed. Through the analysis of its physiological and biochemical indicators, the influence of drought stress on the functions of grassland ecosystems was explored.

### 2.2. Experimental Design

The experiment was conducted at Qingdao Agricultural University. In mid-March 2023, *Festuca elata*, *Lolium perenne*, and *Poa pratensis* were sown separately or mixed in the ratios shown in Table 1. The proportion of mixed broadcasting refers to Yılmaz’s [36] research and selects the common proportion in the local area. Seeding was conducted at a rate of 20 g/m^2^, and each grass mixture ratio was sown in triplicate to ensure experimental reliability. They were uniformly sown in flowerpots (upper radius = 30 cm, lower radius = 20 cm, height = 25 cm), whose cultivation substrate consisted of garden soil and sand mixed in a 1:1 ratio and the cultivation was carried out [37]. Each different turfgrass configuration was designated A through F, and each of the six treatments was divided into two groups: a Group 1 serving as the control group (without stress) and a Group 2 incurring the drought stress treatment. Once the plants had grown into a turf-like shape, the experimental research began. Each group was replicated three times.

Before the experiment, all pots were watered sufficiently to reach a saturated soil water content, after which only the control group was replenished with water (daily). Each experimental group was subjected to continuous drought by stopping the water supply, and soil water content change in the pots was monitored with a soil moisture meter (HH2 moisture meter delta-t devices, Agriexpo). Sampling and measurements were carried out every 7 days. The drought stress group was supplemented with water after 21 days of no water. In the preliminary experiment before the formal experiment, we found that when plants begin to wither, but do not die in large numbers, the soil water content is between 2% and 5%. Therefore, when the soil water content of the drought stress group was measured daily at below 5%, an appropriate water supplementation of 20–100 mL was carried out [38].

The daily amount of dust fall in Qingdao is about 3.2 t/km^2^·30 d. Artificial dust raising was therefore carried out to simulate this dust fall rate. Sampling and measurement of the corresponding physiological and ecological variables was undertaken at 0 d, 7 d, 14 d, 21 d, and recovery 14 days (expressed as 35 d) during the recovery phase.

The location of the experiment was selected as Qingdao Agricultural University and its surrounding areas. According to the characteristics of land use types and vegetation distribution in Qingdao City, Shandong Province, the area along the roads around Qingdao Agricultural University was selected as the study area. Twelve sampling points were set up, with each monitoring point spaced 300 m apart. During the sampling process, it is necessary to ensure that the structures of shrubs and grasses near each sample point are as consistent as possible. A dust collection box with a diameter of 20 cm and a depth of 15 cm was selected and fixed to a tree trunk 1 m above the ground to and collect air dust samples. Three sampling stations were set up at each sampling point to collect sand and dust samples from different sources. The collected dust was screened through a microporous filter membrane with a pore size of 10 μm to obtain the final dust sample [39].

**Figure 1:**
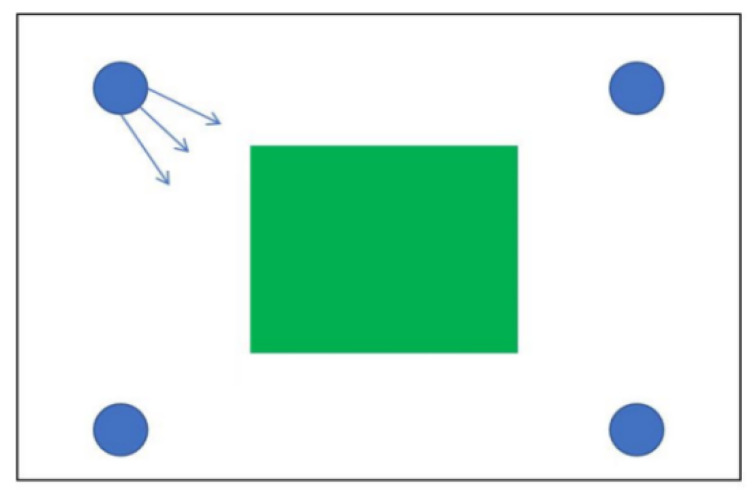
Schematic diagram of the locus.

**Figure 2:**
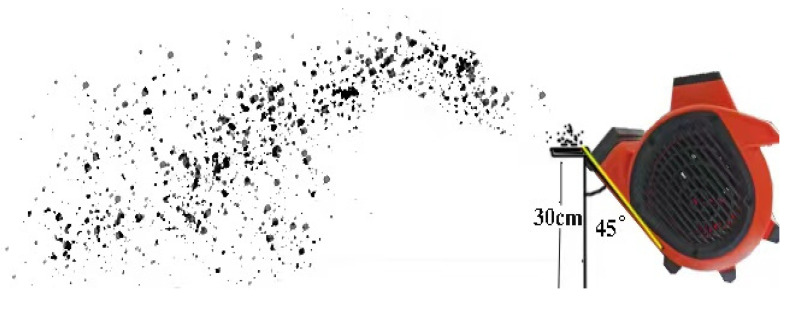
Schematic diagram of artificial dust raising.

Before the experiment, a portable sprayer was used to rinse the plant leaves 2 to 3 times to remove dust particles adhering to the leaf surface, so as to prevent the previously accumulated dust from affecting the test results. The plants were placed in an artificial dust suppression chamber measuring 4.5 m by 3 m. Each plant configuration was set up for three repetitions to carry out the artificial dust suppression experiment. Manual dust suppression was carried out using a blower (SK-800B, Supercloud). A blower was installed on the bracket. A piece of paper measuring 10 cm × 20 cm was parallel to the floor and fixed at the air outlet at a 45° angle to the blower. The wind speed was set at 1000 r/min. The dust was blown towards the plants with the blower (Figure 1 and Figure 2).

### 2.3. Measurement Indexes and Methods

Leaf dust retention, turf appearance quality (TQ), chlorophyll (Chl) content, soluble sugar (Sol) content, malondialdehyde (MDA) content, proline (Pro) content, relative water content (RWC), peroxidase (POD), and superoxide (SOD) dismutase content were measured.

Chlorophyll content was determined by the acetone method; soluble sugar content was determined by the anthrone method; malondialdehyde was determined by the thiobarbituric acid method; proline content was determined by the acidic indole-3-acetic acid colorimetric method; SOD activity was determined by the nitro blue tetrazolium method; and POD activity was the determined by guaiacol method [40,41,42,43,44,45].

The NTEP nine-level scoring method was adopted to evaluate the quality of the tested grass plants under drought stress conditions. The National Turf Evaluation Program of the United States, abbreviated as NTEP, is a means used to test grasslands throughout the United States. This plan has received joint support from the United States Department of Agriculture, the United States Agricultural Service, and the National Grassland Foundation. Since 1980, this assessment method has begun to evaluate the growth status of grasslands under different environmental conditions, different maintenance and management measures, and different application conditions.

The NTEP score is an appearance quality scoring method that takes into account the color, structure, density, uniformity, and overall quality of the lawn. The scoring criteria of NTEP are divided into 9 points. A score of 9 is the best score that a meadow can achieve, and 1 means that a meadow has withered or is in a dormant state. This experiment is scored on a 9-point scale. A score of 1–2 indicates dormant or semi-dormant grassland, 2–4 indicates very poor quality, 4–5 indicates poor quality, 5–6 indicates average quality, 6–7 indicates good, 7–8 indicates excellent, and above 8 indicates extremely excellent.

Dust retention measurement [46]: After placing the leaves in distilled water and letting them stand for 2 h, they were rinsed with a small amount of distilled water. The rinse solution was first filtered with a dried and weighed microporous membrane (dried and weighed, W1), whose pore size was 10 μm. Next, this filter membrane was oven-dried to a constant weight, and the weight of the dried filter membrane was weighed, as W2. The total dust retention was then calculated as W = W2 − W1.

### 2.4. Data Analysis

Excel 2016 software was used for data statistics and organization, while SPSS 26 was used for the statistical analysis. Measurements are expressed as the mean value ± SD (standard deviation), and the group difference in means analyzed by ANOVA (one-way analysis of variance). Correlation analysis is carried out using Pearson correlation. Graphpad 9 was used for plotting the results. The comprehensive evaluation was carried out by the membership function method.

## 3. Results

### 3.1. The Changes in the Physiological and Ecological Variables of Six Groups of Lawn Grasses Under Drought Stress

#### 3.1.1. The Influence of Drought Stress on the TQ of Lawn Grass

**Figure 3:**
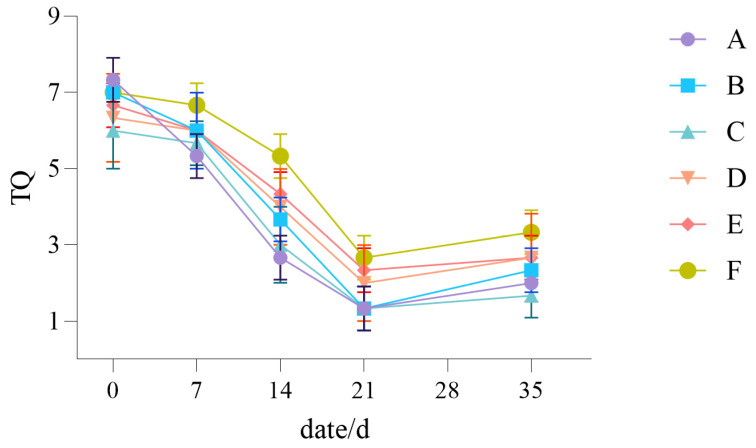
Turf quality of the six turfgrass treatments (Group A–F).

The NTEP nine-point rating method was applied to evaluate the impact of drought stress on the TQ of the tested turfgrass. As Figure 3 shows, the overall trend of TQ for the six different turfgrass configuration groups is consistent. With more days of drought, the stress it induces increases and the turf’s appearance quality decreases. In the early stage of drought stress, the TQ of the six turfgrass treatments decreased slightly, but after 14 days (as indicated in Figure 3, 21d—35d), the decline was significantly sharper, at which point Group A, Group B, and Group C declined to below a score of 4. The order of TQ scores for the six turfgrass treatments, from high to low, was Group F > Group E > Group D > Group B > Group C > Group A.

#### 3.1.2. The Influence of Drought Stress on the RWC of Lawn Grass

**Figure 4:**
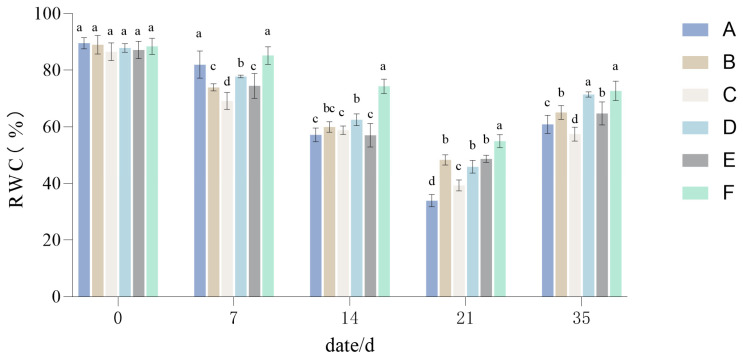
Changes in the relative water content (RWC) of the six different turfgrass treatments (Group A–F). Different lowercase letters indicate significant differences between the six different lawn grass groups (*p* < 0.05).

Under drought stress, the overall RWC showed a downward trend and was lowest at 21 days (Figure 4). Among the configuration groups, compared with their RWC at 0 days, Group A decreased by 55.60%, Group B decreased by 40.65%, Group C decreased by 47.23%, Group D decreased by 41.96%, Group E decreased by 48.73%, and Group F decreased by 33.42%.

After 21 days, water was replenished for the drought-stressed group, and the RWC was measured at 35 days. The RWCs of different groups recovered to some extent. Compared with RWC at the end of the drought treatment (the 21-day mark), Group A increased by 26.95%, Group B increased by 16.74%, Group C increased by 18.15%, Group D increased by 25.52%, Group E increased by 16.01%, and Group F increased by 17.72%. From Figure 4, it can also be seen that although RWC did rebound with water replenishment, it did not quite reach its initial level before the drought treatment; after rewatering, the RWCs in the different treatments differed significantly from their initial value, with Group A rising by 28.65%, and likewise Group B by 23.92%, Group C by 29.08%, Group D by 16.44%, Group E by 22.31%, and Group F by 15.70%. Hence, the different treatments varied by more than two-fold in their recovery ability after experiencing drought, ranked as follows: Group F > Group D > Group E > Group B > Group A > Group C.

#### 3.1.3. The Influence of Drought Stress on the Chl Content of Lawn Grass

**Figure 5:**
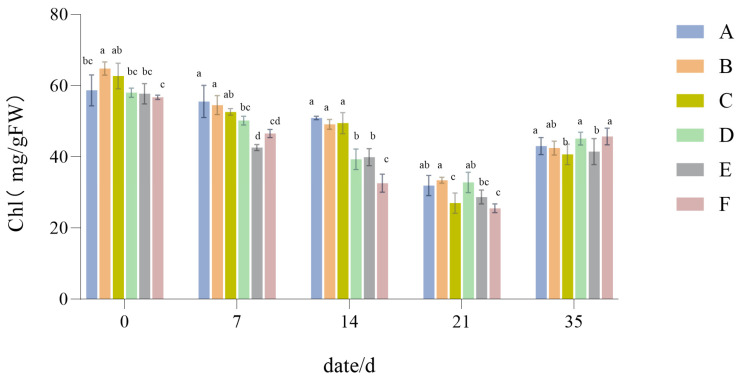
Chlorophyll (Chl) content results. Different lowercase letters indicate significant differences in Chl content between the six different turfgrass treatments (*p* < 0.05).

As the duration of drought continued, the Chl content showed a trend of decreasing, but the degree of change depended on the type of configuration. From Figure 5, it is evident that in the early stage of drought, Chl decreased to differing extents, with Group C undergoing the largest reduction.

After 21 days of recovery and water replenishment, the Chl content of each configuration increased to some extent, but failed to return to its initial level. As seen in Figure 5, the difference between the initial value and recovered value is smallest for Group F, indicating that it possessed a stronger ability to recover from drought-induced damage.

#### 3.1.4. The Influence of Drought Stress on the Sol Content of Lawn Grass

**Figure 6:**
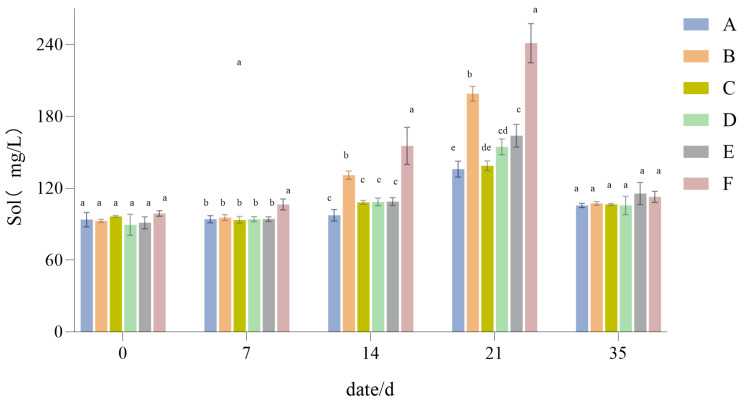
Soluble sugar (Sol) content results. Different lowercase letters indicate significant differences in Sol content between the six different turfgrass treatments (*p* < 0.05).

From Figure 6, it can be seen that the Sol content rose with prolonged drought, this increase being largest for Group F and smallest for both Group A and Group C. Therefore, Group F accumulated a large amount of Sol under the imposed drought stress. After 21 days of recovery and water replenishment, the content of Sol fell rapidly and returned to a normal level after a period of time (Figure 6).

#### 3.1.5. The Influence of Drought Stress on the Pro Content of Lawn Grass

**Figure 7:**
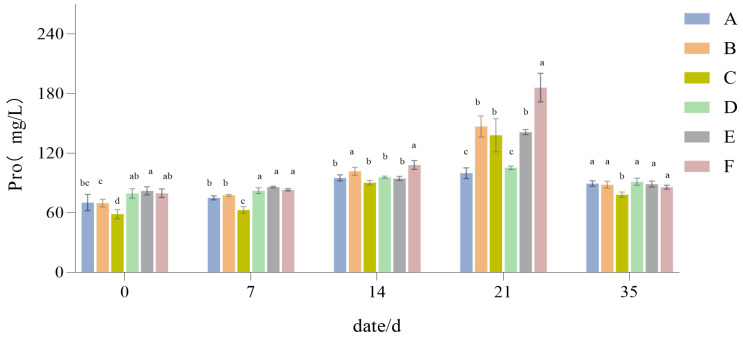
Proline (Pro) content results. Different lowercase letters indicate significant difference in the Pro content between the six different turfgrass treatments (*p* < 0.05).

With continued drought, the Pro content of the different treatments significantly increased (Figure 7), proving that drought stress led to the accumulation of Pro in turfgrass. However, Figure 7 also shows that the magnitude of Pro increase in the different treatments was not the same, being significantly higher in Group F than the other five groups. When the stress reached 21 days, in Group A and Group D, compared with the other groups, Pro was still increasing, but it was not significant. After 21 days of recovery and with water replenishment till the 35-day mark, the content of Pro gradually returned to its initial (pre-drought) levels.

#### 3.1.6. The Influence of Drought Stress on the MDA Content of Lawn Grass

**Figure 8:**
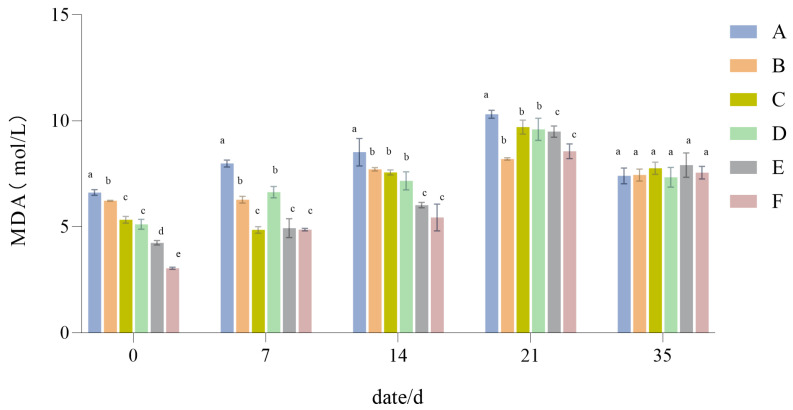
Malondialdehyde (MDA) content results. Different lowercase letters indicate significant differences in MDA content between the six different turfgrass treatments (*p* < 0.05).

As seen in Figure 8, the MDA content of the six configuration groups generally increased with the degree of drought. However, that increase varied, with Group F increasing the most, to 5.51 mol/L, and Group B increasing the least, to 1.97 mol/L. Furthermore, at 21 days, Group A had the highest MDA content, and was most damaged by drought stress; conversely, Group B had the lowest MDA content, indicating that it was least damaged by drought stress. After 14 days of recovery and water replenishment, the MDA content was reduced in all groups, but did not reach its initial (pre-drought) levels, for which Group B differed the least from its initial value.

#### 3.1.7. The Influence of Drought Stress on the POD Activity of Lawn Grass

**Figure 9:**
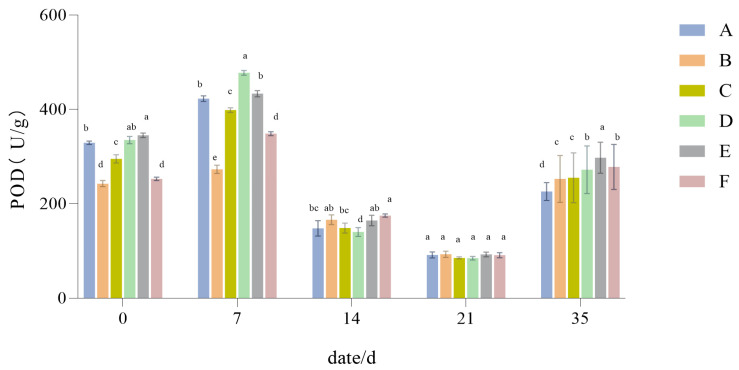
Peroxidase activity (POD). Different lowercase letters indicate significant POD activity between the six different turfgrass treatments (*p* < 0.05).

As shown in Figure 9, the POD activity of the different treatments rose at the onset of drought, and then decreased significantly at high stress levels. With longer drought, the POD activity levels of the six treatments all fell, with activity levels ranked from large to small as follows: Group F > Group B > Group D > Group E > Group A > Group C. After receiving a normal supply of water, by the 35-day mark, the POD activity in all treatments had rebounded and gradually returned to its normal levels (*p* < 0.05).

#### 3.1.8. The Influence of Drought Stress on the SOD Activity of Lawn Grass

**Figure 10:**
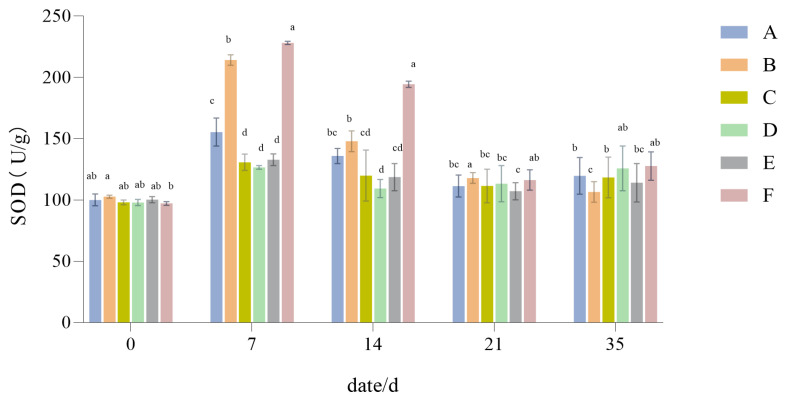
Superoxide dismutase activity (SOD). Different lowercase letters indicate significant SOD activity between the six different turfgrass treatments (*p* < 0.05).

From Figure 10, it is evident that SOD activity showed a trend of first increasing and then decreasing, with Groups B and F showing significantly higher SOD activity than the other turfgrass configuration groups. After 21 days of recovery and water replenishment till 35 days, the SOD activity of the different treatments had gradually returned to near-normal levels (*p* < 0.05).

#### 3.1.9. Changes in Dust Retention

**Figure 11:**
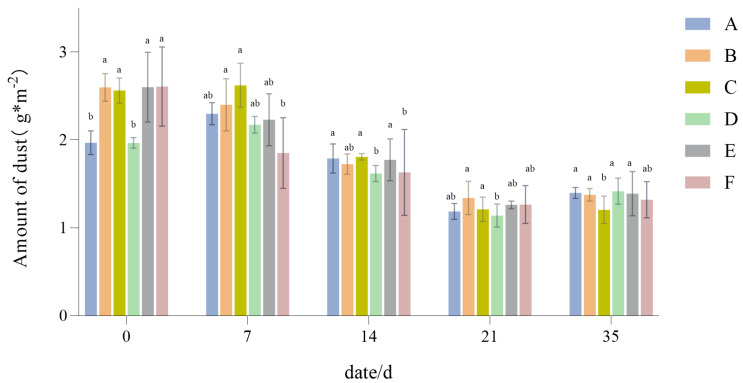
Changes in dust retention in the six different turfgrass treatments (A–F). Different lowercase letters indicate significant differences between the six different lawn grass groups (*p* < 0.05).

From Figure 11, it can be seen that the dust retention capacities of the different turfgrass treatments under drought stress all showed a trend of increasing at first and then decreasing. In the early stage of drought (0 to 7 days), the dust retention performance of the drought-stressed group increased slightly, but as drought continued it decreased rapidly, such that even after the recovery phase it could not regain its initial value.

### 3.2. Data Analysis

#### 3.2.1. Correlation Analysis of the Effects of Drought Stress on Physiological Indicators of Lawn Grass

**Figure 12:**
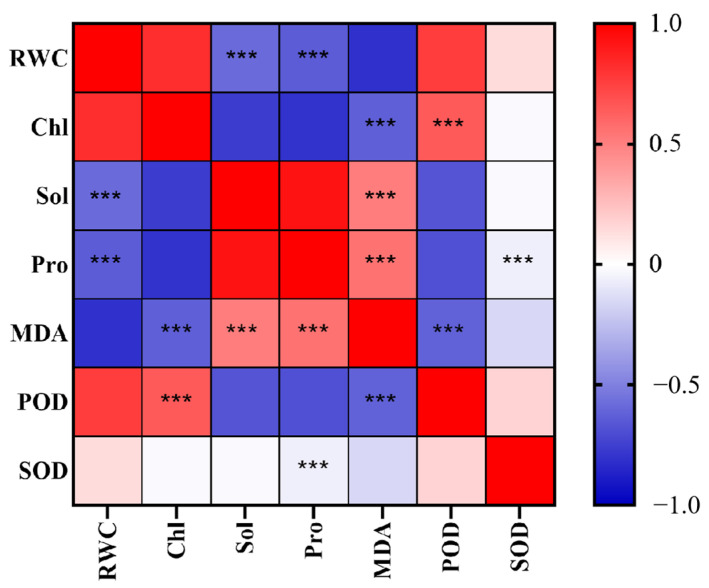
Correlation heat map of impacts of drought stress on physiological indexes of turfgrass. “***” represents significant differences.

Bivariate correlations were tested for the seven variables: RWC, Chl, Sol, Pro, MDA, POD, and SOD (Table 2). From Figure 12, it can be seen that RWC was significantly negatively correlated with Sol and Pro; Chl was significantly negatively correlated with MDA yet positively correlated with POD; Sol was significantly negatively correlated with RWC but positively correlated with MDA; Pro was significantly negatively and positively correlated with RWC and MDA, respectively; MDA was significantly negatively correlated with both Chl and POD, though significantly positively correlated with both Sol and Pro; POD was significantly positively correlated with Chl while negatively correlated with MDA; and SOD was weakly correlated with Pro, albeit significantly.

#### 3.2.2. Membership Function Analysis of Drought Resistance of Turfgrass in Six Treatments

Using the membership function method of fuzzy mathematics, the seven physiological and ecological variables of the six different turfgrass treatments were comprehensively evaluated for drought resistance. An average value of each configuration’s membership function was then obtained (Table 3): the larger its value, the stronger the drought resistance. Among the groups, the 2:6:2 configuration exhibited the highest average value, at 0.7543, whereas that of *Poa pratensis* was the lowest, 0.2454. The data indicated that the drought resistance of the six different turfgrass treatments took this rank order: Group F > Group B > Group E > Group D > Group A > Group C.

#### 3.2.3. Principal Component Analysis (PCA) of the Effects of Drought Stress on Physiological Indicators of Lawn Grass

**Figure 13:**
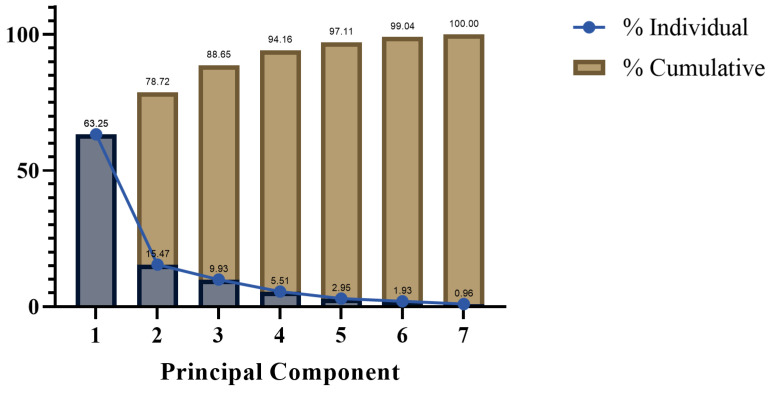
Proportion of variance explained in the PCA.

**Figure 14:**
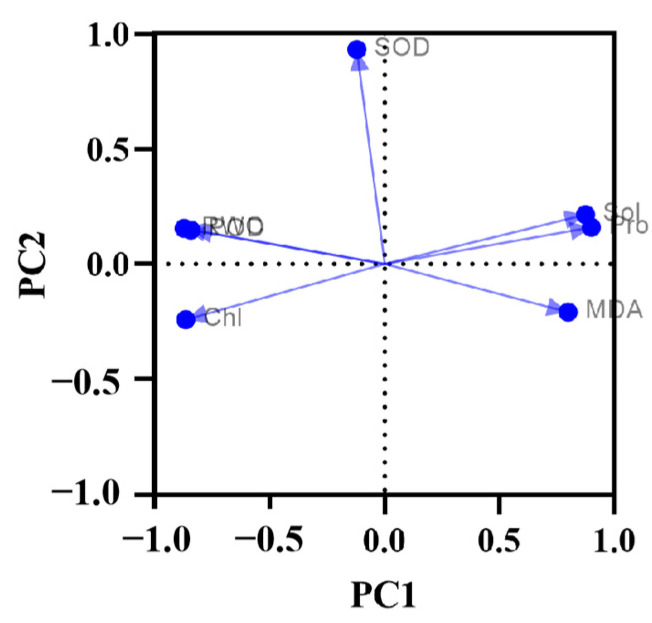
Loadings from the PCA.

PCA was then performed on the experimental data. From Figure 13, it can be seen that the first component explained 63.25% of the total variance found in the variables, and the second component explains an additional 15.47%, together explaining 78.72%. In the loading diagram of Figure 14, the closer any two variables are, the stronger their positive correlation coefficient, and the higher their positive correlation. At the same time, Figure 14 also displays the grouping of the data columns for the first two principal components. Evidently, RWC and POD show a similar pattern, and likewise for Sol and Pro, which have a stronger positive correlation. Figure 14 also reveals that PC1 is negatively correlated with POD, SOD, Chl, and RWC, yet positively correlated with Pro, Sol, and MDA. Meanwhile, PC2 is negatively correlated with POD, SOD, RWC, Sol, and Pro, though positively correlated with both MDA and Chl.

**Figure 15:**
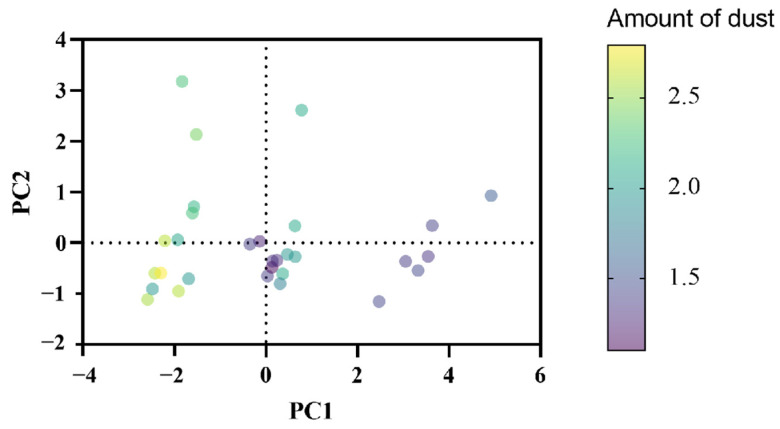
PCA scores.

It can be seen from Figure 15 that the dust retention amount is mainly distributed along the *X*-axis. On the *Y*-axis, there are only a few additional points on the periphery, and it is clear that the dust retention amount decreases as PC1 increases. It can be seen from Figure 14 that MDA, Pro, and Sol show a positive correlation with PC1. Therefore, these three indicators have a negative correlation effect on the amount of dust retention. The sample points are distributed in a relatively scattered manner throughout the coordinate system and do not present an obvious linear or nonlinear aggregation structure, indicating that the data has a certain diversity in the directions of these two principal components after dimension reduction.

The data points are predominantly clustered in the upper-left and lower regions. The upper-left region exhibits relatively lower dust retention levels with dense data point distribution, while the lower region shows higher dust retention with more dispersed data points.

Most data points are concentrated in the lower-left quadrant, indicating that samples in this area share similar characteristics regarding the studied variables. Along the PC2 axis, this region demonstrates a clear trend: as PC2 values decrease, dust retention capacity increases. This pattern is corroborated by Figure 14, which reveals a negative correlation between Chl content and PC2—higher Chl levels correspond to greater dust retention.

These PCA results align with experimental observations where both Chl content and dust retention displayed elevated values during the initial drought stage (Figure 5 and Figure 11), creating mutual validation between the multivariate analysis and experimental measurements.

## 4. Discussion

Through research and comprehensive evaluation, it is possible to screen treatments of turfgrass for better drought resistance and dust retention ability.

### 4.1. The Responses of TQ of Six Groups of Lawn Grasses to Drought Stress

In the drought treatment, among the six turfgrass treatments, the *Lolium perenne* (Group A) has the lowest turf appearance quality and RWC. Under drought stress conditions, the trend of change in the leaves’ RWC is the same as that for their TQ. The less water in leaves, the lower the appearance quality, the greater the drought damage to the turfgrass plants, and thus, the weaker their drought resistance. In the experiment, the RWC of *Lolium perenne* monoculture decreased the most, indicating that its drought resistance is the weakest; however, the decrease was smallest in the 2:6:2 mix configuration, indicating that its drought resistance is the strongest overall.

### 4.2. The Responses of Chlorophyll Content of Six Groups of Lawn Grasses to Drought Stress

When turfgrass first faces drought stress, their chlorophyll content shows an increasing trend, indicating that the plants have an adaptive regulatory mechanism under drought stress conditions. This regulatory mechanism acts to avoid drought-induced damage via various metabolic reactions so as to maximize drought resistance, a finding that is consistent with that of Wang et al. [47]. However, as the drought lengthens, the chlorophyll content will gradually decrease, which is consistent with the reported findings of Subhash et al. [48].

Among the six configuration groups, the chlorophyll content of the 2:6:2 mix of *Lolium perenne*, *Festuca elata*, and *Poa pratensis* was the least affected, while that of the *Poa pratensis* monoculture was the most affected. Under drought stress, *Poa pratensis* exhibited a substantial increase in MDA levels (from approximately 5 μmol/mL to 10 μmol/mL) and a significant decrease in POD activity (from about 4500 U/g·min to 1000 U/g·min). These observations suggest that *Poa pratensis* likely has a diminished capacity to scavenge reactive oxygen species (ROS). The consequent accumulation of ROS may induce oxidative damage to the photosynthetic apparatus, disrupting the structure and function of chlorophyll molecules. This mechanistic explanation accounts for the pronounced decline in chlorophyll content observed in *Poa pratensis* monocultures.

After recovery and water replenishment, the chlorophyll content increased to some extent, but did not reach the initial level. Among the groups, the recovered value of the 2:6:2 mix configuration differed least from its initial value, indicating that it has a better recovery ability after damage.

### 4.3. The Responses of Osmotic Regulatory Substances and Antioxidation of Six Groups of Lawn Grasses to Drought Stress

Proline has an osmotic regulatory role in the drought resistance of turfgrass and is also a protective and adaptive response under drought stress [49]. The proline content in turfgrass increased rapidly with a longer duration of drought stress, and plants with strong drought resistance accumulated more proline. During the experiment, the accumulation amount of proline was positively correlated with the drought resistance of different treatments, a result consistent with findings of Iqbal et al. [50]. The 2:6:2 mix of *Lolium perenne*, *Festuca elata*, and *Poa pratensis* had a higher accumulation of proline during the drought stress process (*p* < 0.05), indicating that its drought resistance is relatively strong; the proline accumulation of the *Lolium perenne* monoculture and the 2:3:5 mix was relatively low in comparison with the other treatments (*p* < 0.05), but always higher than the other monocultures, indicating that they have a certain drought resistance. It also shows that proline plays a prominent role in the water retention of plants and improving their resistance to drought stress.

The content of soluble sugar is a protective regulatory mechanism enabling turfgrass to adapt to a drought environment [51]. Under drought stress conditions, the soluble sugar content was significantly higher in the 2:6:2 mix than in the other treatments, suggesting that it has a better regulatory ability. In tandem, the rate of increase in soluble sugar was also significantly faster in that particular mix, indicating that its soluble sugar regulatory effect gradually strengthens with prolonged drought stress. This response would be beneficial for maintaining normal water metabolism in a drought environment, in line with the results of Bhusal et al. [52]. After restoring the normal water supply, the content of soluble sugar in each configuration fell rapidly and regained its normal levels after a period of time; for example, there was little change in Group A, while there was a significant decrease in Group F (decreased from around 60 mg/mL to around 20 mg/mL). Among the treatments, the rate of decrease in the 2:6:2 mix was fastest, implying that it is more sensitive to changes in water and harbors a better recovery ability post-damage.

The more malondialdehyde that accumulates, the greater the damage incurred by a plant [53]. During the drought stress process, the malondialdehyde contents of the six different turfgrass treatments all demonstrated an increasing trend. Among them, the increase in the malondialdehyde content of the *Festuca elata* monoculture was the smallest, indicating that this configuration type is the least damaged by drought; the increase in the 2:6:2 mix was the largest, suggesting that it is affected more by drought. Nevertheless, after 21 days of recovery and water replenishment, when compared with the other groups, the decrease in malondialdehyde content was greater in the 2:6:2 mix, indicating that it has a stronger recovery ability after drought disturbances.

Plants under drought stress incur impaired cell stability and oxidative damage [54]. During the experiment, the levels of POD and SOD activity exhibited a trend of increasing at first and then decreasing with a longer period of drought. Hanslin et al. [55] found that plant cells have various mechanisms to clear out specific reactive oxygen species (ROS), among which the antioxidant mechanisms of POD and SOD are closely linked and form a potent antioxidant system. Accordingly, since the POD and SOD activity in *Festuca elata* and the 2:6:2 mix configuration is higher, their antioxidant capacity is likely stronger, and so the impact of drought on them is smaller.

### 4.4. The Responses of the Dust Retention Abilities of Six Groups of Lawn Grasses to Drought Stress

Drought will affect the leaf morphology, growth and development, and photosynthetic efficiency of plants [56], which may affect their dust retention ability in urban areas [57]. The present study’s experiment shows that the dust retention of different turfgrass treatments under drought stress eventually tends to decline, falling rapidly after 7 days, mainly for several reasons. First, the leaves of the drought-stressed group exhibited signs of yellowing after 7 days, and their leaf area was reduced significantly. After many leaves are shed, gas turbulence within the plant will be weakened, thereby reducing the number of collisions between coarse particles and the plant’s surface [58], leading to a rapid decrease in dust retention ability. Second, despite some water replenishment provided in the later stage of drought to prevent the plant from dying, only a few green leaves persisted around the target plant at 21 days; this means the sample almost exclusively has young and newly grown leaves, while mature leaves are rougher and more likely to capture atmospheric particles [59]. Third, after drought stress, the wettability and viscosity of the plant leaf surface are both greatly reduced, which will weaken its retention of intercepted airborne particles [20]. The wind generated by artificial dust raising will also cause some of the particles retained on the surface to become resuspended into the air. Although the effect of wind was avoided in the experiment, the resuspension effect of particulate matter is still enhanced by the generated wind [60].

The experimental results show that the dust retention performance of turfgrass exhibits a trend of increasing in the early stage of drought. Based on this and the other results, two reasons for this are plausible. First of all, plants regulate the opening of their leaf stomata to adapt to environmental changes via evaporation, and this water vapor repels particles away from the stomatal area [61]. In the early stage of drought, turfgrass can regulate the opening of stomata in the short-term to adapt to environmental changes [62], but since that evaporation eventually decreases, there is a greater deposition of particles on the leaf surface. Secondly, plants under drought treatment have more salt particles on the stomatal upper surface, which is consistent with the results of Räsänen et al. [25]. These salt particles will augment the surface roughness of turfgrass, thereby inducing particles to settle more effectively on the leaves of drought-treated turfgrass. In summary, due to reduction in stomatal conductance caused by soil drought, water’s evaporation from plants is reduced, enabling more particles to be deposited on the turfgrass leaves. Therefore, in practical applications, to adapt to shifting climatic conditions and trap more particulate matter from air, the soil water content of turfgrass can be manipulated (appropriately reduced). When the duration or severity of drought is controlled within a certain range, a higher dust retention ability provisioned by turfgrass has more environmental benefits, especially in urban and semi-urban areas.

### 4.5. The Shortcomings of the Experiment

This study investigated the physiological responses and dust retention capacity changes of different turfgrass mixtures under sustained drought conditions, but certain limitations should be noted.

Firstly, the research was conducted using pot experiments rather than field trials. As demonstrated by Laio [63], who established an analytical framework for soil moisture dynamics across different soil depths based on vertical water distribution profiles, along with subsequent studies by Laio et al. [64] and Tamea et al. [65] which examined soil moisture dynamics in natural environments, the restricted soil depth in pot experiments leads to fundamentally different water movement patterns compared to actual turfgrass applications.

Furthermore, this study utilized road traffic dust as the experimental material, but in reality, the types of dust faced by turfgrass vary significantly across different urban functional zones. Research has shown [66] that turfgrass in urban industrial areas is subjected to more severe dust pollution stress—dust in these zones (such as near power plants, incinerators, and various industrial facilities) contains higher concentrations of heavy metals (including Pb, Cu, Zn, and Cd), volatile organic compounds (VOCs), and polycyclic aromatic hydrocarbons (PAHs) [67,68]. Industrial activities have been identified as one of the primary anthropogenic sources of airborne dust pollution [69,70]. However, this study did not include experiments targeting the dust types typical of industrial areas.

To conclude, the findings of this study demonstrate applicability potential under controlled experimental conditions, but inherent limitations (e.g., pot-based system, standardized dust composition) may affect direct extrapolation to real-world scenarios.

## 5. Conclusions

On the 21st day of the drought stress treatment, the turfgrass appearance quality, leaf relative water content, chlorophyll content, proline content, malondialdehyde content, soluble sugar content, POD activity, SOD activity, and dust retention were significantly higher in the 2:6:2 mix configuration of *Lolium perenne*, *Festuca elata*, and *Poa pratensis* in comparison with other configuration groups. Through a comprehensive analysis of their drought resistance, the six turfgrass treatments can be ranked from strongest to weakest, as follows: 2:6:2 mix > *Festuca elata* > 2:2:6 mix > 2:3:5 mix > *Lolium perenne* > *Poa pratensis*, while their ability to recover after drought stress takes this order (from strongest to weakest): 2:6:2 mix > 2:3:5 mix > 2:2:6 mix > *Festuca elata* monoculture > *Lolium perenne* monoculture > *Poa pratensis* monoculture. Their dust retention ability under drought stress is *Festuca elata* > 2:6:2 mix > 2:2:6 mix > *Poa pratensis* > *Lolium perenne* > 2:3:5 mix. The 2:6:2 mix demonstrated optimal drought resistance, recovery capacity, and comprehensive physiological responses under drought stress while maintaining effective dust retention, making it the recommended configuration for turf establishment in arid regions.

In future studies, it will be necessary to investigate plants’ dust capture capacity for different dust sources across various regions, while also transitioning from pot experiments to field trials to enhance the generalizability of research findings to real-world conditions.

## Figures and Tables

**Table 1:** Details of the six different turfgrass treatments.

Turfgrass Species and Proportion				
Codename for the Mix		*Lolium perenne*	*Festuca elata*	*Poa pratensis*
Group A	A1	10	0	0
	A2	10	0	0
Group B	B1	0	10	0
	B2	0	10	0
Group C	C1	0	0	10
	C2	0	0	10
Group D	D1	2	3	5
	D2	2	3	5
Group E	E1	2	2	6
	E2	2	2	6
Group F	F1	2	6	2
	F2	2	6	2

**Table 2:** Correlation coefficients of impacts of drought stress on physiological indexes of turfgrass.

	RWC	Chl	Sol	Pro	MDA	POD	SOD
RWC	1	0.8187	−0.5869	−0.6404	−0.8155	0.7574	0.1345
Chl	0.8187	1	−0.7668	−0.8069	−0.6310	0.6457	−0.0271
Sol	−0.5869	−0.7668	1	0.9285	0.5095	−0.6741	−0.0217
Pro	−0.6404	−0.8069	0.9285	1	0.5509	−0.688	−0.0648
MDA	−0.8155	−0.6311	0.5095	0.5509	1	−0.6226	−0.1567
POD	0.7574	0.6457	−0.6741	−0.6881	−0.6226	1	0.1716
SOD	0.1345	−0.0271	−0.0217	−0.0648	−0.1567	0.1716	1

**Table 3:** Comprehensive evaluation of the membership function of six groups of lawn grass treatments on drought resistance.

Group	A	B	C	D	E	F
RWC	0.19063	0.33385	0	0.53069	0.32391	1
Chl	0.88625	1	0.68068	0.49266	0.08918	0
Sol	0	0.52411	0.08904	0.13611	0.24904	1
Pro	0.01415	0.48894	0	0.22715	0.56265	1
MDA	0	0.43768	0.49434	0.43838	0.72375	1
POD	0.62159	0	0.50791	0.92349	1	0.38569
SOD	0.25931	0.61044	0.02905	0	0.00039	1
Average	0.2817	0.485007	0.25729	0.39264	0.42127	0.76938
sequence	5	2	6	4	3	1

## Data Availability

The data that support the findings of this study are available from the corresponding author upon reasonable request.
